# Decline in Handgrip Strength From Midlife to Late-Life is Associated With Dementia in a Japanese Community: The Hisayama Study

**DOI:** 10.2188/jea.JE20180137

**Published:** 2020-01-05

**Authors:** Yozo Hatabe, Mao Shibata, Tomoyuki Ohara, Emi Oishi, Daigo Yoshida, Takanori Honda, Jun Hata, Shigenobu Kanba, Takanari Kitazono, Toshiharu Ninomiya

**Affiliations:** 1 Department of Epidemiology and Public Health, Graduate School of Medical Sciences, Kyushu University, Fukuoka, Japan; 2 Department of Neuropsychiatry, Graduate School of Medical Sciences, Kyushu University, Fukuoka, Japan; 3 Center for Cohort Studies, Graduate School of Medical Sciences, Kyushu University, Fukuoka, Japan; 4 Department of Medicine and Clinical Science, Graduate School of Medical Sciences, Kyushu University, Fukuoka, Japan

**Keywords:** Alzheimer’s disease, dementia, epidemiology, handgrip strength, longitudinal study

## Abstract

**Background:**

The association between decline in handgrip strength from midlife to late life and dementia is unclear.

**Methods:**

Japanese community-dwellers without dementia aged 60 to 79 years (ie, individuals in late life; mean age, 68 years) were followed for 24 years (1988–2012) (*n* = 1,055); 835 of them had participated in a health examination in 1973–1974 (mean age, 53 years), and these earlier data were used for the midlife analysis. Using a Cox proportional hazards model, we estimated the risk conferred by a decline in handgrip strength over a 15-year period (1973–74 to 1988) from midlife to late life on the development of total dementia, Alzheimer’s disease (AD), and vascular dementia (VaD) over the late-life follow-up period from 1988 to 2012.

**Results:**

During the follow-up, 368 subjects experienced total dementia. The age- and sex-adjusted incidence of total dementia increased significantly with greater decline in handgrip strength (increased or unchanged handgrip strength [≥+0%] 25.1, mildly decreased [−14 to −1%] 28.4, and severely decreased [≤−15%] 38.9 per 1,000 person-years). A greater decline in handgrip strength was significantly associated with higher risk of total dementia after adjusting for potential confounding factors; subjects with severely decreased handgrip strength had 1.51-fold (95% confidence interval, 1.14–1.99, *P* < 0.01) increased risk of total dementia compared to those with increased or unchanged handgrip strength. Similar significant findings were observed for AD, but not for VaD.

**Conclusions:**

Our findings suggest that a greater decline in handgrip strength from midlife to late life is an important indicator for late-life onset of dementia.

## INTRODUCTION

Accumulating epidemiological evidence suggests that maintaining higher levels of physical activity is important to prevent premature death, disability, and cognitive impairment in late life.^1^^–^^4^ We previously reported the relationship between late life physical activity and the development of dementia in a Japanese elderly population.^5^ Physical activity can be estimated using several measures of physical performance, such as handgrip strength.^6^^,^^7^ Handgrip strength is an easy, non-invasive, and inexpensive measure of muscle strength in the elderly, which has been reported to be well-correlated with the muscle strength of limbs and the human trunk.^8^^,^^9^ Additionally, it has been reported that lower handgrip strength is associated with a greater risk of diabetes mellitus,^10^ cardiovascular disease, all-cause and cardiovascular mortality, and physical function and frailty.^11^^,^^12^ Several population-based studies have also shown that reduced handgrip strength in late life was associated with increased risks of cognitive impairment and dementia.^13^^–^^17^ These findings raise the possibility that maintaining handgrip strength, as well as systemic muscle strength, from midlife to late life would be important for preventing late-life risk of dementia, because these muscle strengths begin to decrease with age after midlife.^18^ However, there is no evidence regarding the association between midlife handgrip strength or its change from midlife to late life and the risk of dementia.

The aim of this study was to investigate the influence of handgrip strength measured in midlife and late life and its change with time on the development of dementia and its subtypes in a general elderly population.

## METHODS

### Study sample

**Figure 1.**
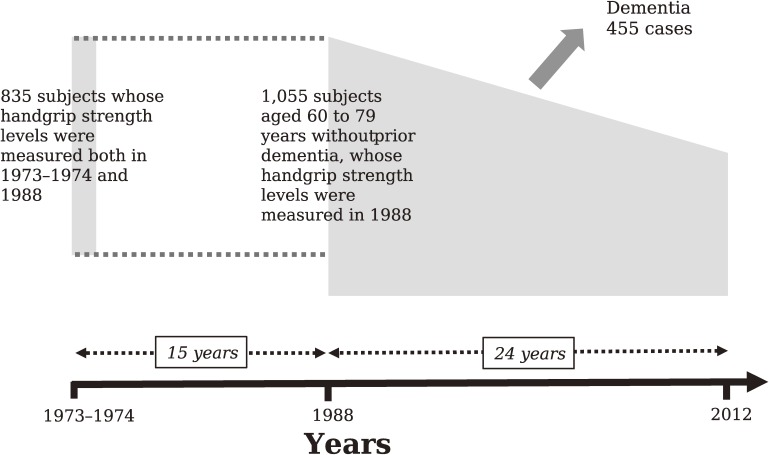
Diagram of the study design

The Hisayama study is a prospective cohort study of cerebrocardiovascular diseases that was begun in 1961 in the town of Hisayama, a suburban community adjacent to the metropolitan area of Fukuoka, Japan.^18^^,^^19^ Full community surveys on the health and neurological status of residents aged 40 years or more have been repeated every 1 to 2 years since 1961.^8^^,^^19^ Additionally, comprehensive surveys of cognitive impairment in the elderly have also been conducted every 6 or 7 years since 1985.^19^^,^^20^ In 1988, 1,072 residents aged 60 to 79 years (89.6% of the total population in this age group) participated in a health examination survey for this study. After excluding 13 participants with dementia and 4 participants whose handgrip strength was not measured, the remaining 1,055 participants (449 men and 606 women) were enrolled in an analysis investigating the association between late-life handgrip strength and the development of dementia and its subtypes. Additionally, 835 of these subjects who had also participated in a health checkup survey conducted in 1973–1974 (age of participants in this survey: 45–64 years) were included in an analysis examining the association between midlife handgrip strength or the change of handgrip strength from midlife to late life and the risk of late-life dementia (Figure 1).

This study was approved by the Kyushu University Institutional Review Board for Clinical Research. We obtained written informed consent from the participants.

### Follow-up survey

The participants were followed-up prospectively for 24 years, from December 1988 to November 2012 (mean 14.6; standard deviation [SD], 7.3 years). The methods for screening potential dementia events were described previously.^19^^,^^21^ In summary, information about new events, including stroke and cognitive impairment, was collected through a daily monitoring system established by the study team, local physicians, and members of the town’s Health and Welfare Office. Regular health examinations, including physical and neurological examinations, were also repeated every year to obtain information on new events of stroke and dementia missed by the monitoring system. Health information was checked annually via letter or telephone for any participants who did not undergo regular examination or who had moved out of town. Additionally, in order to precisely detect dementia cases to the greatest extent possible, comprehensive assessment of cognitive function, including neuropsychological tests, such as the Mini-Mental State Examination^22^ and the Hasegawa’s Dementia Scale Revised version,^23^ was performed in 1992, 1998, 2005, and 2012. Subjects suspected of having new neurological symptoms, including cognitive impairment, were evaluated by the study team. Additionally, when a subject died, we reviewed all the available clinical information, interviewed the attending physician and the family of the deceased, and tried to obtain permission for autopsy from the family.

### Diagnosis of dementia

The guidelines of the Diagnostic and Statistical Manual of Mental Disorders, Revised Third Edition were used to define the diagnosis of dementia.^24^ The criteria of the National Institute of Neurological and Communicative Disorders and Stroke and the Alzheimer’s Disease and Related Disorders Association were used to make a diagnosis of Alzheimer’s disease (AD),^25^ and the criteria of the National Institute of Neurological Disorders and Stroke-Association International pour la Recherche et l’Enseignementen Neuroscience were used to define subjects with vascular dementia (VaD).^26^ Clinical information, including neuroimaging, was used to diagnose possible or probable dementia subtypes. Definite dementia subtypes were also determined based on clinical and neuropathological information. The diagnostic procedure for autopsy cases has been reported previously.^27^ A neuropathological diagnosis of AD was made following the National Institute on Aging-Reagan Institute criteria,^28^ where the frequency of neuritic plaques and neurofibrillary tangles was evaluated using the Consortium to Establish a Registry for Alzheimer’s Disease criteria^29^ and Braak stage.^30^ Definite VaD cases were confirmed to have causative stroke or cerebrovascular change and no neuropathological evidence of other forms of dementia. Expert stroke physicians and psychiatrists adjudicated every dementia case.

During the 24-year follow-up period, 368 participants (44.0%; 120 men and 248 women) developed total dementia. Of these, 328 (89.1%) received evaluation with brain imaging and 192 (52.2%) underwent a brain autopsy; in 175 cases both were performed. Thus, 345 subjects in all (93.5%) had some kind of morphological examination. Among dementia cases, 34 cases were a mixed type of AD and VaD and were counted as events in the analysis for each subtype. In summary, 218 participants experienced AD and 107 participants VaD.

### Assessment of handgrip strength

The handgrip strength of subjects was measured by a public health nurse using a Smedley Hand Dynamometer (MIS, Tokyo, Japan). The width of the handle was adjusted such that the second phalanx was against the inner stirrup. The participants were encouraged to exert maximal handgrip strength. Handgrip strength was measured twice for each hand alternately, and the maximum value among four measurements was used for the analyses in both 1973–1974 and 1988.^11^

### Other risk factors

In both the 1973–1974 and 1988 surveys, each participant completed a self-administered questionnaire on educational status, medical history, anti-hypertensive treatment, smoking habits, and alcohol consumption. Educational status was categorized as ≤6 years of formal education. Smoking habits and alcohol consumption were classified as either current use or not. The information on regular exercise was also collected using a self-administered questionnaire in 1988, with participants engaging in sports at least three times a week during their leisure time being defined as the regular exercise group, but data on regular exercise were not available in 1973–1974. History of stroke was determined as the previous occurrence of a sudden onset of nonconvulsive and focal neurological deficit persisting for >24 hours on the basis of all available clinical data according to the Classification of Cerebrovascular Disease III (CVD-III) criteria.^31^

Blood pressure was measured three times with the subject in a sitting position, with a mercury sphygmomanometer at the right upper arm after at least 5 minutes of rest in both surveys; the mean of the three measurements was used for the analysis. Body height and weight were measured in light clothing without shoes, and body mass index (kg/m^2^) was calculated in both surveys. Electrocardiogram abnormalities were defined as left ventricular hypertrophy (Minnesota code 3-1), ST segment depression (4-1, 2, or 3), or atrial fibrillation (8-3) in both surveys. Diabetes was defined by fasting glucose levels ≥7.0 mmol/L, postprandial glucose levels ≥11.1 mmol/L, and/or medical history of diabetes in 1973–1974, and it was defined by the administration of antidiabetic treatment, plasma glucose levels (fasting glucose level ≥7.0 mmol/L or postprandial glucose level ≥11.1 mmol/L), or a 75 g oral glucose tolerance test using the 1998 World Health Organization criteria in 2008. Plasma glucose levels were measured by the glucose-oxidase method. Serum total cholesterol levels were measured using the Zurkowski method in 1973–1974 and an enzymatic autoanalyzer in 1988.

### Statistical analyses

The participants were divided into three groups (ie, low, medium, and high) based on age- and sex-specific tertiles of handgrip strength, where the age-groups were categorized by 5-year intervals in order to control for the confounding caused by rapid decrease in the handgrip strength with aging (Table 1). The linear trend of the mean values or frequencies of risk factors across handgrip strength levels were tested using linear or logistic regression analysis. The incidences of dementia and its subtypes were calculated using the person-year method. The hazard ratio (HR) and its 95% confidence interval (CI) of dementia and its subtypes across the tertiles of handgrip strength measured at the survey in 1988 (late life) or in 1973–1974 (midlife) were estimated using a Cox proportional hazards model with adjustment for potential confounding factors collected at each survey—namely, age, sex, education level, systolic blood pressure, use of antihypertensive agents, diabetes mellitus, total cholesterol, BMI, electrocardiogram abnormalities, smoking habit, alcohol intake, and regular exercise (only for the analysis of late life). The proportional hazards assumption was checked graphically using the log cumulative hazard plot for outcomes according to the levels of handgrip strength. Additionally, we investigated the influence of the percentage change rate of handgrip strength from midlife to late life, which was calculated as [(handgrip strength in 1988/handgrip strength in 1973–1974) − 1] * 100, on the risk of dementia. We categorized the %change of handgrip strength as follows. First, subjects with a %change of 0% or higher were classified into an “increased or unchanged” group, and those with a %change under 0% were classified into a decreased group. Then, we divided the decreased group into a mildly decreased (−14 to −1%) and a severely decreased (≤−15%) subgroup using a cutoff value of 15%, which was chosen based on a report that muscle strength decreases annually by about 1% (ie, 15% over 15 years) after 50 years of age.^32^^,^^33^ The age- and sex-adjusted cumulative incidence of outcomes across the %change of handgrip strength levels was estimated based on the regression estimates from a Cox proportional hazards model including age and sex. The heterogeneity in the relationship between subgroups (ie, sexes or age groups) was tested by adding interaction terms to the relevant Cox model. We also performed sensitivity analyses as follows; the analysis adding impaired glucose tolerance (defined as a fasting glucose level <7.0 mmol/L and 2-hour postprandial glucose level of 7.8 to 11.0 mmol/L) as an adjustment variable, the analysis excluding subjects with a history of stroke, the analysis by using other cut-off values for the %change of handgrip strength, such as median values of the decreased group or the “increased or unchanged” group, and the analysis using a Fine and Gray model. All statistical analyses were performed using SAS 9.4 software (SAS Institute, Cary, NC, USA). Two-sided *P* < 0.05 was considered statistically significant in all analyses.

**Table 1.** Ranges of the tertiles of handgrip strength measured in 1988 or 1973–1974 according to age and sex group

Men					Women				
	
Age, years	Average handgrip strength, kg	Tertiles of handgrip strength, kg			Age, years	Average handgrip strength, kg	Tertiles of handgrip strength, kg		
	
		Low	Medium	High			Low	Medium	High
***For the analysis of late life handgrip strength measured in 1988***									
60–64 years	38.3	<36.5 (*n* = 61)	36.5–39.5 (*n* = 50)	>39.5 (*n* = 73)	60–64 years	22.9	<21.0 (*n* = 56)	21.0–24.5 (*n* = 72)	>24.5 (*n* = 77)
65–69 years	36.2	<33.0 (*n* = 32)	33.0–38.5 (*n* = 37)	>38.5 (*n* = 36)	65–69 years	20.2	<18.5 (*n* = 54)	18.5–21.5 (*n* = 44)	>21.5 (*n* = 71)
70–74 years	33.2	<31.0 (*n* = 30)	31.0–35.5 (*n* = 29)	>35.5 (*n* = 34)	70–74 years	17.9	<16.0 (*n* = 39)	16.0–19.5 (*n* = 42)	>19.5 (*n* = 50)
75–79 years	29.3	<27.0 (*n* = 21)	27.0–31.5 (*n* = 23)	>31.5 (*n* = 23)	75–79 years	17.1	<15.0 (*n* = 28)	15.0–19.0 (*n* = 33)	>19.0 (*n* = 40)

***For the analysis of midlife handgrip strength measured in 1973–1974***									
45–49 years	38.6	<36.0 (*n* = 47)	36.0–40.5 (*n* = 46)	>40.5 (*n* = 49)	45–49 years	24.5	<22.0 (*n* = 45)	22.0–26.0 (*n* = 60)	>26.0 (*n* = 59)
50–54 years	36.5	<33.5 (*n* = 23)	33.5–38.5 (*n* = 28)	>38.5 (*n* = 26)	50–54 years	22.3	<20.5 (*n* = 44)	20.5–24.0 (*n* = 43)	>24.0 (*n* = 48)
55–59 years	35.1	<33.5 (*n* = 23)	33.5–36.5 (*n* = 19)	>36.5 (*n* = 30)	55–59 years	21.6	<20.0 (*n* = 29)	20.0–22.5 (*n* = 37)	>22.5 (*n* = 34)
60–64 years	32.9	<30.5 (*n* = 19)	30.5–35.0 (*n* = 20)	>35.0 (*n* = 20)	60–64 years	21.5	<19.0 (*n* = 24)	19.0–23.0 (*n* = 33)	>23.0 (*n* = 29)

## RESULTS

Table 1 shows the age- and sex-stratified tertiles of handgrip strength in late life (1988) and midlife (1973–1974). Both in late life and midlife, the handgrip strength of both sexes decreased significantly with aging, and the average handgrip strength of women was significantly lower than that of men regardless of age group.

The clinical characteristics of the study population by handgrip strength levels in late life and midlife are summarized in Table 2. In late life, the mean values of diastolic blood pressure, serum total cholesterol, and BMI increased significantly with higher levels of handgrip strength, while subjects with higher handgrip strength were significantly less likely to have diabetes mellitus and history of stroke. In midlife, the mean values of BMI and diastolic blood pressure increased significantly with higher handgrip strength.

**Table 2.** Characteristics of participants according to handgrip strength levels measured in late life (in 1988) and in midlife (in 1973–1974)

Risk factors at 1988 or 1973–1974	Overall	Handgrip strength levels			*P* for trend

		Low	Medium	High	
***Late life (in 1988)***					
Number of subjects, men/women	449/606	144/177	139/191	166/238	
Age, years	68 (6)	68 (6)	68 (6)	67 (6)	0.20
Men, %	42.6	44.9	42.1	41.1	0.31
Education ≤6 years, %	11.0	12.3	8.2	12.3	0.92
Systolic blood pressure, mm Hg	139 (23)	139 (23)	140 (22)	139 (23)	0.93
Diastolic blood pressure, mm Hg	76 (11)	75 (10)	76 (11)	77 (11)	0.04
Antihypertensive medication, %	25.0	25.2	25.5	24.5	0.81
Diabetes mellitus, %	14.5	17.8	14.2	12.1	0.03
Serum total cholesterol, mmol/L	5.40 (1.13)	5.31 (1.15)	5.36 (1.20)	5.49 (1.06)	0.03
BMI, kg/m^2^	22.4 (3.2)	21.6 (3.2)	22.4 (3.1)	23.0 (3.1)	<0.01
Electrocardiogram abnormalities, %	21.0	23.4	20.6	19.6	0.22
History of stroke, %	5.3	9.0	3.9	3.5	<0.01
Smoking habits, %	29.1	32.2	29.7	25.9	0.09
Alcohol consumption, %	27.5	30.8	25.7	26.3	0.22
Regular exercise ≥3 times/wk, %	14.3	11.8	14.5	16.1	0.11
***Midlife (in 1973–1974)***					
Number of subjects, men/women	350/485	112/142	113/173	125/170	
Age, years	53 (6)	53 (6)	53 (6)	54 (6)	0.45
Men, %	41.9	44.1	39.5	42.4	0.72
Education ≤6 years, %	10.9	12.2	11.5	9.1	0.25
Systolic blood pressure, mm Hg	134 (22)	131 (22)	135 (22)	135 (22)	0.06
Diastolic blood pressure, mm Hg	78 (12)	76 (11)	78 (12)	79 (12)	<0.01
Antihypertensive medication, %	5.9	5.5	5.2	6.8	0.51
Diabetes mellitus, %	1.6	1.2	2.1	1.4	0.90
Serum total cholesterol, mmol/L	4.89 (0.84)	4.83 (0.81)	4.94 (0.86)	4.90 (0.83)	0.36
BMI, kg/m^2^	22.4 (3.0)	21.5 (3.1)	22.5 (2.7)	23.1 (3.0)	<0.01
Electrocardiogram abnormalities, %	12.3	14.2	9.8	13.2	0.79
History of stroke, %	0.6	0.0	1.0	0.7	0.34
Smoking habits, %	36.4	36.6	35.7	36.9	0.92
Alcohol consumption, %	29.8	32.3	30.4	27.1	0.18
Regular exercise ≥3 times/wk, %	NA	NA	NA	NA	

BMI, body mass index; NA, not available.

Values are represented as the mean (standard deviation, or frequency).

Next, we estimated the association between late-life and midlife handgrip strength and the risk of developing dementia (Table 3). With respect to late life, there was a significant inverse relationship between late-life handgrip strength and the age- and sex-adjusted HRs of total dementia, AD, and VaD. These associations remained significant even after adjustment for potential confounding factors in 1988. Compared with subjects with high late-life handgrip strength, the multivariable-adjusted HRs of total dementia, AD, and VaD were significantly higher in those with low late-life handgrip strength.

**Table 3.** Hazard ratios for the development of total dementia and its subtypes according to handgrip strength levels in late life or midlife

Handgrip strength levels	Number of events	Number of participants	Person-years at risk	Age- and sex-adjusted			Multivariable-adjusted^a,b^		

				HR	95% CI	*P* value	HR	95% CI	*P* value
***Late life (in 1988)***									
Total dementia									
High	164	404	6,470	1.00	Reference		1.00	Reference	
Medium	145	330	4,833	1.32	1.06–1.66	0.01	1.34	1.04–1.73	0.02
Low	146	321	4,366	1.64	1.31–2.05	<0.01	1.66	1.29–2.13	<0.01
*P* for trend				<0.01			<0.01		
AD									
High	94	404	6,470	1.00	Reference		1.00	Reference	
Medium	88	330	4,833	1.41	1.05–1.89	0.02	1.37	0.99–1.90	0.06
Low	91	321	4,366	1.85	1.39–2.48	<0.01	1.94	1.41–2.67	<0.01
*P* for trend				<0.01			<0.01		
VaD									
High	51	404	6,470	1.00	Reference		1.00	Reference	
Medium	39	330	4,833	1.13	0.74–1.71	0.58	1.22	0.76–1.98	0.41
Low	55	321	4,366	1.84	1.25–2.70	<0.01	2.07	1.32–3.25	<0.01
*P* for trend				<0.01			<0.01		
***Midlife (in 1973–1974)***									
Total dementia									
High	126	295	4,460	1.00	Reference		1.00	Reference	
Medium	122	286	4,395	0.98	0.76–1.25	0.84	0.95	0.74–1.22	0.68
Low	120	254	3,581	1.32	1.03–1.70	0.03	1.29	0.996–1.67	0.05
*P* for trend				0.04			0.07		
AD									
High	74	295	4,460	1.00	Reference		1.00	Reference	
Medium	70	286	4,395	0.94	0.68–1.30	0.71	0.94	0.67–1.30	0.70
Low	75	254	3,581	1.46	1.06–2.01	0.02	1.46	1.05–2.03	0.02
*P* for trend				0.03			0.03		
VaD									
High	37	295	4,460	1.00	Reference		1.00	Reference	
Medium	37	286	4,395	1.03	0.65–1.62	0.90	0.95	0.60–1.51	0.84
Low	33	254	3,581	1.16	0.72–1.86	0.54	1.07	0.66–1.74	0.79
*P* for trend				0.55			0.81		

AD, Alzheimer’s disease; CI, confidence interval; HR, hazard ratio; VaD, vascular dementia.

^a^Adjusted for potential confounding covariates measured in 1988 for the analysis of late life handgrip strength—namely, age, sex, education level, systolic blood pressure, use of antihypertensive agents, diabetes mellitus, total cholesterol, body mass index, electrocardiogram abnormalities, smoking habit, alcohol intake, and regular exercise.

^b^Adjusted for potential confounding covariates measured in 1973–1974 for the analysis of midlife handgrip strength—namely, age, sex, education level, systolic blood pressure, use of antihypertensive agents, diabetes mellitus, total cholesterol, body mass index, electrocardiogram abnormalities, smoking habit, and alcohol intake.

As for midlife, there was a significant inverse relationship between midlife handgrip strength and the age- and sex-adjusted HRs of total dementia and AD. The multivariable-adjusted HRs of AD was significantly higher in subjects with low handgrip strength as compared with those with high handgrip strength, whereas statistically significant association was not detected for total dementia and VaD.

**Figure 2.**
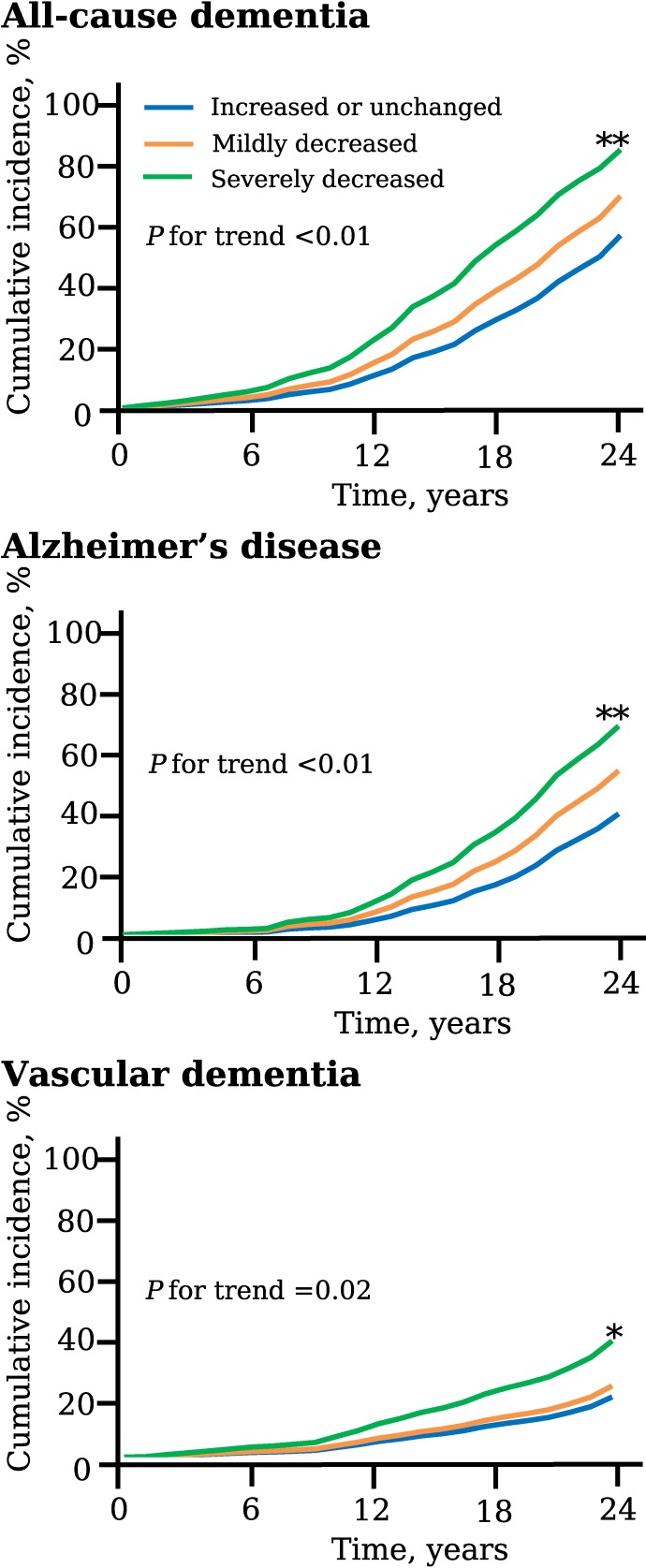
The age- and sex-adjusted cumulative incidence for total dementia and its subtypes according to %change of handgrip strength, the Hisayama Study, 1988–2012. The %change of handgrip strength was divided into three groups: increased or unchanged, ≥+0%; mildly decreased, −14 to −1%; severely decreased, ≤−15%.

Finally, we estimated the association of the %change of handgrip strength from midlife to late life with the risk of dementia. Figure 2 shows the age- and sex-adjusted cumulative incidence for total dementia and its subtypes according to the %change of handgrip strength levels. The age- and sex-adjusted cumulative incidence of total dementia, AD, and VaD increased significantly according to decrement of the %change levels as follows: increased or unchanged, mildly decreased, and severely decreased. The clinical characteristics of the study population by the %change of handgrip strength levels are summarized in Table 4. As shown in Table 5, the age- and sex-adjusted incidence of total dementia, AD, and VaD showed an increasing linear trend with the decrease in handgrip strength. Greater decline in handgrip strength was significantly associated with higher risk of total dementia and AD after adjusting for potential confounding factors. Meanwhile, the multivariable-adjusted risk of VaD also tended to increase with a greater decline in handgrip strength, but the association did not reach statistically significant levels. There was no evidence of heterogeneity in the association between sexes (all *P* for heterogeneity >0.1), or between age groups (60–69 years and 70–79 years) (all *P* for heterogeneity >0.3). We performed the sensitivity analyses by adding impaired glucose tolerance as an adjustment variable (eTable 1), after excluding subjects with a history of stroke (eTable 2), and by using alternative cut-off values of handgrip %change, such as the median value of the decreased group or the median value of the “increased or unchanged” group (eTable 3 and eTable 4). However, these sensitivity analyses did not make any material differences in the findings of Table 3 and Table 5. Finally, the sensitivity analysis using a Fine and Gray model was performed to account for the influence of competing risks of death. As a consequence, the associations of handgrip strength in midlife or late life and %change of handgrip strength with the risk of AD and VaD were attenuated so that some associations did not reach the level of statistical significance, but the findings were not altered substantially (eTable 5).

**Table 4.** Characteristics of participants at baseline (1988) according to the %change of handgrip strength from midlife (1973–1974) to late life (1988)

Risk factors at 1988	Overall	%Change of handgrip strength from midlife to late life^a^			*P* for trend

		Increased or unchanged (≥+0%)	Mildly decreased (−14 to −1%)	Severely decreased (≤−15%)	
Number of subjects, men/women	350/485	164/170	123/145	63/170	
Age, years	68 (6)	67 (5)	68 (6)	69 (6)	<0.01
Men, %	41.9	49.1	45.9	27.0	<0.001
Education ≤6 years, %	10.9	10.5	9.7	12.9	0.41
Systolic blood pressure, mm Hg	138 (22)	136 (22)	138 (22)	140 (23)	0.02
Diastolic blood pressure, mm Hg	76 (11)	76 (11)	76 (11)	75 (11)	0.51
Antihypertensive medication, %	24.3	23.7	21.6	28.3	0.25
Diabetes mellitus, %	13.0	10.8	13.1	15.9	0.08
Serum total cholesterol, mmol/L	5.35 (1.11)	5.35 (1.11)	5.34 (1.11)	5.37 (1.12)	0.86
BMI, kg/m^2^	22.3 (3.1)	22.5 (3.1)	22.1 (2.9)	22.1 (3.3)	0.07
Electrocardiogram abnormalities, %	20.8	19.5	20.1	23.6	0.25
History of stroke, %	4.9	1.8	3.0	11.6	<0.01
Smoking habits, %	27.1	29.7	32.2	18.2	0.01
Alcohol consumption, %	26.4	28.0	28.9	21.4	0.11
Regular exercise ≥3 times/wk, %	14.5	16.8	14.9	10.7	0.05

BMI, body mass index.

Values are represented as the mean (standard deviation, or frequency).

^a^{(handgrip strength in 1988/handgrip strength in 1973–1974) − 1} * 100.

**Table 5.** Hazard ratios for the development of total dementia and its subtypes according to the %change of handgrip strength from midlife (1973–1974) to late life (1988)

%Change of handgrip strength^a^	Number of events	Number of participants	Person-years at risk	Age- and sex-adjusted incidence rate^b^	Age- and sex-adjusted			Multivariable-adjusted^c^		

					HR	95% CI	*P* value	HR	95% CI	*P* value
Total dementia										
Increased or unchanged (≥+0%)	138	334	5,495	25.1	1.00	Reference		1.00	Reference	
Mildly decreased (−14 to −1%)	108	268	3,802	28.4	1.19	0.92–1.53	0.18	1.01	0.75–1.34	0.96
Severely decreased (≤−15%)	122	233	3,139	38.9	1.65	1.28–2.13	<0.01	1.51	1.14–1.99	<0.01
*P* for trend				<0.01	<0.01			0.01		
AD										
Increased or unchanged (≥+0%)	81	334	5,495	14.7	1.00	Reference		1.00	Reference	
Mildly decreased (−14 to −1%)	68	268	3,802	17.9	1.28	0.93–1.78	0.13	1.17	0.81–1.68	0.41
Severely decreased (≤−15%)	70	233	3,139	22.3	1.59	1.14–2.22	<0.01	1.62	1.13–2.31	<0.01
*P* for trend				<0.01	<0.01			<0.01		
VaD										
Increased or unchanged (≥+0%)	41	334	5,495	7.5	1.00	Reference		1.00	Reference	
Mildly decreased (−14 to −1%)	28	268	3,802	7.4	1.03	0.63–1.67	0.91	0.91	0.51–1.60	0.73
Severely decreased (≤−15%)	38	233	3,139	12.1	1.79	1.12–2.85	0.01	1.55	0.91–2.64	0.10
*P* for trend				0.02	0.02			0.12		

AD, Alzheimer’s disease; CI, confidence interval; HR, hazard ratio; VaD, vascular dementia.

^a^{(handgrip strength in 1988/handgrip strength in 1973–1974) − 1} * 100.

^b^Per 1000 person-years.

^c^Adjusted for potential confounding covariates measured in 1988—namely, age, sex, education level, systolic blood pressure, use of antihypertensive agents, diabetes mellitus, total cholesterol, body mass index, electrocardiogram abnormalities, smoking habit, alcohol intake, and regular exercise.

## DISCUSSION

The present study clearly demonstrated that lower handgrip strength in late life was significantly associated with the development of total dementia, AD, and VaD in an elderly Japanese population. Moreover, lower handgrip strength in midlife was also linked with greater risk of AD. Intriguingly, individuals with greater decline in handgrip strength of 15% or more over 15 years had significantly higher risks of total dementia and AD. These findings suggest that maintaining handgrip strength as well as systemic muscle strength after midlife would be of clinical importance in the prevention of late-life dementia.

A number of epidemiologic prospective studies have demonstrated an inverse association between late-life handgrip strength and cognitive function in elderly populations.^34^^–^^37^ A scoping review revealed a clear relationship between lower handgrip strength and the progression of cognitive decline.^17^ Likewise, lower late-life handgrip strength has been reported to be significantly associated with a greater risk of total dementia in several prospective studies of the elderly.^15^^,^^38^^,^^39^ With regard to subtypes of dementia, it has been reported that the risk of AD increased significantly with lower late-life handgrip strength in Asian and Western countries.^15^^,^^38^^,^^39^ These results are consistent with our findings. The present study also showed that lower midlife handgrip strength and greater decline in handgrip strength were significantly linked with increased risk of total dementia and AD. To the best of our knowledge, this is the first prospective cohort study to show a long-term association of midlife handgrip strength or a change of handgrip strength from midlife to late life with the risk of dementia and its subtypes.

The mechanism of the relationship between reduced handgrip strength and the risk of dementia has not been clearly defined, but we can suggest several possible explanations. First, higher handgrip strength may be a proxy for the presence of habitual exercise, which has been reported to be associated with decreased risk of dementia in several epidemiological or clinical studies.^38^^,^^39^ In the present study, subjects whose %change of handgrip strength was increased or unchanged from midlife to late life had a higher percentage of habitual exercise, as shown in Table 4. The habitual exercise promotes the maintenance of greater muscle strength after midlife and an improvement of cardiovascular function, resulting in late-life health benefits to improve cognitive function. Second, lower handgrip strength is one of the indicators of frailty. Frailty is characterized by multi-system impairments, including reduced systemic muscle strength.^40^ A population-based study has reported on the association between frailty and AD pathology.^41^ Frailty and AD pathology share underlying pathogeneses, such as vascular pathology, energy production, and stress. Thus, frailty may be a non-cognitive manifestation of AD pathology before dementia appears. Third, lower handgrip strength may reflect systemic inflammation, which has been reported to be linked to cognitive decline and risk of dementia.^42^^–^^45^ It has been reported that inflammation induces the loss of skeletal muscle.^45^ A population-based study showed a significant relationship between low grip strength and elevated levels of inflammatory markers.^46^

The strengths of our study include the longitudinal population-based design, the long-term follow-up period of 24 years, the evaluation of midlife handgrip strength as well as late-life handgrip strength and change of handgrip strength over 15 years, the accuracy of the diagnosis of dementia and its subtypes on the basis of medical information, and the availability of neuroimaging and morphologic examination of the brains of most dementia cases with autopsy. However, some limitations in this study should be noted. First, there is a possibility of reverse causality in the associations between late-life handgrip strength and dementia. However, the present study revealed that lower handgrip strength in midlife and the greater decline in handgrip strength from midlife to late life were linked with the increased risk of dementia. Therefore, reverse causality was unlikely to exist in the present study. Second, the generalizability of the findings may be limited because the findings were gathered from a single site in Japan. Third, the information on regular exercise and impaired glucose tolerance were unavailable for the analysis of midlife handgrip strength. Finally, there could be residual confounding caused by unmeasured factors, including physical activities, disability status, depressive status, and sleep deprivation.^47^^,^^48^

Taken together, the present results suggest that lower handgrip strength in midlife as well as in late life is an important indicator or a possible interventional target for the late-life onset of total dementia and its subtypes in a general Japanese population. Furthermore, individuals with greater decline in handgrip strength over 15 years should be considered to have strongly increased risks of total dementia and AD. Given that the harmful effects of muscle loss on the risk of dementia are likely to be irreversible, the maintenance of systemic muscle strength as well as handgrip strength after midlife may be an effective approach to preventing late life dementia in the general population.

## APPENDIX A. SUPPLEMENTARY DATA

eTable 1. Adjusted hazard ratios for the development of total dementia and its subtypes according to handgrip strength levels in late-life or percent change of handgrip strength from midlife (1973–1974) to late-life (1988)

eTable 2. Hazard ratios for the development of total dementia and its subtypes according to late-life or midlife handgrip strength levels or percent change of handgrip strength from midlife (1973–1974) to late-life (1988) after excluding subjects with history of stroke

eTable 3. Hazard ratios for the development of total dementia and its subtypes according to the %change of handgrip strength from midlife (1973–1974) to late-life (1988), in which the categories of mildly and severely decreased handgrip strength were divided into the median value of percent change of handgrip strength among the subjects assigned in either category

eTable 4. Hazard ratios for the development of total dementia and its subtypes according to the %change of handgrip strength from midlife (1973–1974) to late-life (1988), in which the categories of increased or unchanged handgrip strength were divided into the median value of %change of handgrip strength among the subjects assigned in either category

eTable 5. Hazard ratios for composite endpoint of dementia subtypes according to handgrip strength levels in late-life or midlife
