# Supplementary material for: Decline in Handgrip Strength From Midlife to Late-Life is Associated With Dementia in a Japanese Community: The Hisayama Study
Source: J Epidemiol. 2020 Jan 5;30(1):15–23. doi: 10.2188/jea.JE20180137 (PMC6908846; doi:10.2188/jea.JE20180137)
Supplement: Supplementary file 1 [file je-30-015-s001.pdf]

eTable 1. Adjusted hazard ratios for the development of total dementia and its subtypes according to handgrip strength levels in late-life or percent change of handgrip strength from midlife (1973–1974) to late-life (1988)

	Multivariable-adjusted [a]		
	HR	95% CI	*P* value
Late-life handgrip strength levels (in 1988)			
Total dementia			
High	1.00	Reference	
Medium	1.34	1.04–1.73	0.02
Low	1.66	1.29–2.13	<0.01
P for trend	<0.01		
AD			
High	1.00	Reference	
Medium	1.37	0.99–1.91	0.06
Low	1.96	1.42–2.69	<0.01
P for trend	<0.01		
VaD			
High	1.00	Reference	
Medium	1.19	0.74–1.93	0.47
Low	1.99	1.27–3.11	<0.01
P for trend	<0.01		
**Percent change ratio (from 1973–1974 to 1988) [a)]**			
Total dementia			
Increased or unchanged (≥ +0%)	1.00	Reference	
Mildly decreased (-14 to -1%)	1.01	0.75–1.34	0.96
Severely decreased (≤ -15%)	1.51	1.14–1.99	<0.01
P for trend	<0.01		
AD			
Increased or unchanged (≥ +0%)	1.00	Reference	
Mildly decreased (-14 to -1%)	1.28	0.93–1.78	0.13
Severely decreased (≤ -15%)	1.59	1.14–2.22	<0.01
P for trend	<0.01		
VaD			
Increased or unchanged (≥ +0%)	1.00	Reference	
Mildly decreased (-14 to -1%)	0.89	0.50–1.57	0.69
Severely decreased (≤ -15%)	1.51	0.89–2.56	0.13
P for trend	0.15		

AD, Alzheimer’s disease; CI, confidence interval; VaD, vascular dementia.

[a] Adjusted for potential confounding covariates measured in 1988 for the analysis of late-life handgrip strength—namely, age, sex, education level, systolic blood pressure, use of antihypertensive agents, impaired glucose tolerance, diabetes mellitus, total cholesterol, body mass index, electrocardiogram abnormalities, smoking habit, alcohol intake, and regular exercise.

eTable 2. Hazard ratios for the development of total dementia and its subtypes according to late-life or midlife handgrip strength levels or percent change of handgrip strength from midlife (1973–1974) to late-life (1988) after excluding subjects with history of stroke

Handgrip strength levels	Number of events	Number of participants	Person-years at risk	Age- and sex-adjusted			Multivariable-adjusted [a, b]		
				HR	95% CI	*P* value	HR	95% CI	*P* value
Late-life handgrip strength levels (in 1988) [a]									
Total dementia									
High	150	383	6,470	1.00	Reference		1.00	Reference	
Medium	135	312	4,833	1.28	1.01–1.62	0.04	1.25	0.96–1.62	0.097
Low	135	304	4,366	1.61	1.28–2.04	<0.01	1.61	1.24–2.08	<0.01
P for trend				<0.01			< 0.01		
AD									
High	88	383	6,470	1.00	Reference		1.00	Reference	
Medium	84	312	4,833	1.35	1.003–1.83	0.048	1.23	0.88– 1.71	0.23
Low	92	304	4,366	1.93	1.44– 2.59	<0.01	1.90	1.38– 2.62	<0.01
P for trend				<0.01			<0.01		
VaD									
High	44	383	6,470	1.00	Reference		1.00	Reference	
Medium	33	312	4,833	1.06	0.68–1.67	0.79	1.14	0.68–1.93	0.62
Low	44	304	4,366	1.65	1.08–2.52	0.02	1.89	1.16–3.09	0.011
P for trend				0.02			0.012		
Midlife handgrip strength levels (in 1973–1974) [b]									
Total dementia									
High	125	293	4,460	1.00	Reference		1.00	Reference	
Medium	120	284	4,395	0.97	0.75–1.24	0.79	0.94	0.73–1.21	0.63
Low	119	253	3,581	1.32	1.02–1.69	0.03	1.28	0.99–1.67	0.06
P for trend				0.04			0.07		
AD									
High	73	293	4,460	1.00	Reference		1.00	Reference	
Medium	68	284	4,395	0.93	0.67–1.29	0.65	0.92	0.66–1.29	0.63
Low	75	253	3,581	1.48	1.07–2.04	0.02	1.48	1.06–2.06	0.02
P for trend				0.02			0.03		
VaD									
High	36	293	4,460	1.00	Reference		1.00	Reference	
Medium	37	284	4,395	1.06	0.67–1.67	0.82	0.98	0.67–1.79	0.92
Low	33	253	3,581	1.19	0.74–1.91	0.47	1.09	0.61–1.55	0.73
P for trend				0.48			0.74		
**Percent change ratio (from 1973–1974 to 1988)** [a]									

Total dementia									
Increased or unchanged (≥ +0%)	132	328	5,495	1.00	Reference		1.00	Reference	
Mildly decreased (-14 to -1%)	103	260	3,802	1.18	0.91–1.54	1.02	1.02	0.76–1.37	0.89
Severely decreased (≤ -15%)	106	206	3,139	1.55	1.19–2.03	<0.01	1.44	1.08–1.92	0.013
P for trend				<0.01			0.02		
AD									
Increased or unchanged (≥ +0%)	77	328	5,495	1.00	Reference		1.00	Reference	
Mildly decreased (-14 to -1%)	66	260	3,802	1.29	0.93–1.80	0.13	1.17	0.81–1.69	0.41
Severely decreased (≤ -15%)	68	206	3,139	1.65	1.18–2.32	<0.01	1.65	1.15–2.36	<0.01
P for trend				<0.01			<0.01		
VaD									
Increased or unchanged (≥ +0%)	39	328	5,495	1.00	Reference		1.00	Reference	
Mildly decreased (-14 to -1%)	23	260	3,802	0.90	0.54–1.52	0.70	0.90	0.50–1.64	0.73
Severely decreased (≤ -15%)	28	206	3,139	1.47	0.88–2.45	0.14	1.28	0.72–2.29	0.41
P for trend				0.19			0.46		

AD, Alzheimer’s disease; CI, confidence interval; HR, hazard ratio; VaD, vascular dementia.

[a] Adjusted for potential confounding covariates measured in 1988 for the analysis of late-life handgrip strength—namely, age, sex, education level, systolic blood pressure, use of antihypertensive agents, diabetes mellitus, total cholesterol, body mass index, electrocardiogram abnormalities, smoking habit, alcohol intake, and regular exercise.

[b] Adjusted for potential confounding covariates measured in 1973-1974 for the analysis of midlife handgrip strength—namely, age, sex, education level, systolic blood pressure, use of antihypertensive agents, diabetes mellitus, total cholesterol, body mass index, electrocardiogram abnormalities, smoking habit, and alcohol intake.

eTable 3. Hazard ratios for the development of total dementia and its subtypes according to the %change of handgrip strength from midlife (1973–1974) to late-life (1988), in which the categories of mildly and severely decreased handgrip strength were divided into the median value of percent change of handgrip strength among the subjects assigned in either category

Percent change ratio of handgrip strength [a]	Number of Events	Number of participants	Person-years at risk	Age- and sex-adjusted incidence rate [b]	Age- and sex-adjusted			Multivariable-adjusted [c]		
					HR	95% CI	*P* value	HR	95% CI	*P* value
Total dementia										
Increased or unchanged (≥ +0%)	138	334	5,495	25.1	1.00	Reference		1.00	Reference	
Mildly decreased (-13.7 to -1%)	102	250	3,638	28.0	1.16	0.90–1.51	0.25	0.99	0.74–1.33	0.93
Severely decreased (≤ -13.6%)	128	251	3,303	38.8	1.66	1.29–2.14	<0.01	1.50	1.14–1.98	<0.01
P for trend				<0.01	<0.01			<0.01		
AD										
Increased or unchanged (≥ +0%)	81	334	5,495	14.7	1.00	Reference		1.00	Reference	
Mildly decreased (-13.7 to -1%)	64	250	3,638	17. 6	1.26	0.90–1.75	0.18	1.13	0.78–1.64	0.52
Severely decreased (≤ -13.6%)	74	251	3,303	22.4	1.61	1.16–2.24	<0.01	1.63	1.15–2.32	<0.01
P for trend				<0.01	<0.01			<0.01		
VaD										
Increased or unchanged (≥ +0%)	41	334	5,495	7.5	1.00	Reference		1.00	Reference	
Mildly decreased (-13.7 to -1%)	26	250	3,638	7.1	0.99	0.60–1.62	0.96	0.89	0.50–1.59	0.70
Severely decreased (≤ -13.6%)	40	251	3,303	12.1	1.81	1.15–2.88	0.01	1.54	0.91–2.61	0.11
P for trend				0.02	0.02			0.12		

AD, Alzheimer’s disease; CI, confidence interval; HR, hazard ratio; VaD, vascular dementia.

[a] { (handgrip strength in 1988 / handgrip strength in 1973–1974)-1}*100

[b] Per 1,000 person-years

[c] Adjusted for potential confounding covariates measured in 1988—namely, age, sex, education level, systolic blood pressure, use of antihypertensive agents, diabetes mellitus, total cholesterol, body mass index, electrocardiogram abnormalities, smoking habit, alcohol intake, and regular exercise.

eTable 4. Hazard ratios for the development of total dementia and its subtypes according to the %change of handgrip strength from midlife (1973–1974) to late-life (1988), in which the categories of increased or unchanged handgrip strength were divided into the median value of %change of handgrip strength among the subjects assigned in either category

Percent change ratio of handgrip strength [a]	Number of Events	Number of participants	Person-years at risk	Age- and sex-adjusted incidence rate [b]	Age- and sex-adjusted			Multivariable-adjusted [c]		
					HR	95% CI	*P* value	HR	95% CI	*P* value
Total dementia										
Increased (≥ +7%)	72	167	2,883	25.0	0.91	0.65–1.28	0.59	1.08	0.74–1.57	0.71
Unchanged (+7% to 0%)	66	167	2,612	25.3	1.00	Reference		1.00	Reference	
Mildly decreased (-15 to -1%)	108	268	3,802	28.4	1.13	0.83–1.54	0.43	1.04	0.74–1.48	0.81
Severe decrease (≤ -15%)	122	233	3,139	38.9	1.57	1.16–2.14	<0.01	1.56	1.11–2.19	<0.01
P for trend				<0.01	<0.01			0.02		
AD										
Increased (≥ +7%)	41	167	2,883	14.2	0.88	0.57–1.36	0.57	0.96	0.58–1.57	0.86
Unchanged (+7% to 0%)	40	167	2,612	15.3	1.00	Reference		1.00	Reference	
Mildly decreased (-15 to -1%)	68	268	3,802	17.9	1.20	0.81–1.78	0.36	1.14	0.74–1.76	0.55
Severely decreased (≤ -15%)	70	233	3,139	22.3	1.49	1.01–2.22	0.047	1.58	1.03–2.42	0.04
P for trend				<0.01	<0.01			0.015		
VaD										
Increased (≥ +7%)	20	167	2,883	6.9	0.76	0.41–1.40	0.37	1.19	0.58–2.46	0.63
Unchanged (+7% to 0%)	21	167	2,612	8.0	1.00	Reference		1.00	Reference	
Mildly decreased (-15 to -1%)	28	268	3,802	7.4	0.89	0.50–1.57	0.69	0.99	0.50–1.98	0.98
Severely decreased (≤ -15%)	38	233	3,139	12.1	1.55	0.90–2.69	0.12	1.70	0.88–3.30	0.11
P for trend				0.02	0.02			0.24		

AD, Alzheimer's disease; CI, confidence interval; HR, hazard ratio; VaD, vascular dementia.

[a] { (handgrip strength in 1988 / handgrip strength in 1973–1974)-1}*100

[b] Per 1,000 person-years

[c] Adjusted for potential confounding covariates measured in 1988—namely, age, sex, education level, systolic blood pressure, use of antihypertensive agents, diabetes mellitus, total cholesterol, body mass index, electrocardiogram abnormalities, smoking habit, alcohol intake, and regular exercise.

eTable 5. Hazard ratios for composite endpoint of dementia subtypes according to handgrip strength levels in late-life or midlife

	Number of events	Number of participants	Ordinary Cox model			Fine and Gray model		
			HR	95% CI	*P* value	HR	95% CI	*P* value
**AD**								
Late-life handgrip strength levels (in 1988) [a]								
High	94	404	1.00	Reference		1.00	Reference	
Medium	88	330	1.37	0.99–1.90	0.06	1.23	0.90–1.55	0.22
Low	91	321	1.94	1.41–2.67	<0.01	1.52	1.20–1.83	<0.01
Midlife handgrip strength levels (in 1973–1974) [b]								
High	74	295	1.00	Reference		1.00	Reference	
Medium	70	286	0.94	0.67–1.30	0.70	0.97	0.65–1.30	0.86
Low	75	254	1.46	1.05–2.03	0.02	1.25	0.93–1.57	0.18
**%Change ratio (from 1973–1974 to 1988)** [a]								
Increased or unchanged (≥ +0%)	81	334	1.00	Reference		1.00	Reference	
Mildly decreased (-14 to -1%)	68	268	1.17	0.81–1.68	0.41	1.03	0.67–1.39	0.87
Severely decreased (≤ -15%)	70	233	1.62	1.13–2.31	<0.01	1.32	0.96–1.68	0.13
**VaD**								
Late-life handgrip strength levels (in 1988) [a]								
High	51	404	1.00	Reference		1.00	Reference	
Medium	39	330	1.22	0.76–1.98	0.41	1.06	0.58–1.54	0.81
Low	55	321	2.07	1.32–3.25	<0.01	1.69	1.23–2.14	0.02
Midlife handgrip strength levels (in 1973–1974) [b]								
High	37	295	1.00	Reference		1.00	Reference	
Medium	37	286	0.95	0.60–1.51	0.84	0.98	0.53–1.44	0.94
Low	33	254	1.07	0.66–1.74	0.79	1.05	0.56–1.54	0.85
**%Change ratio (from 1973–1974 to 1988)** [a]								
Increased or unchanged (≥ +0%)	41	334	1.00	Reference		1.00	Reference	
Mildly decreased (-14 to -1%)	28	268	0.91	0.51–1.60	0.73	0.79	0.21–1.36	0.42
Severely decreased (≤ -15%)	38	233	1.55	0.91–2.64	0.10	1.32	0.79–1.86	0.31

AD, Alzheimer's disease; CI, confidence interval; HR, hazard ratio; VaD, vascular dementia.

[a] Adjusted for potential confounding covariates measured in 1988 for the analysis of late-life handgrip strength—namely, age, sex, education level, systolic blood pressure, use of antihypertensive agents, diabetes mellitus, total cholesterol, body mass index, electrocardiogram abnormalities, smoking habit, alcohol intake, and regular exercise.

[b] Adjusted for potential confounding covariates measured in 1973–1974 for the analysis of midlife handgrip strength—namely, age, sex, education level, systolic blood pressure, use of antihypertensive agents, diabetes mellitus, total cholesterol, body mass index, electrocardiogram abnormalities, smoking habit, and alcohol intake.
